# Effects of a multi-enzyme complex on growth performance, nutrient utilization and bone mineralization of meat duck

**DOI:** 10.1186/s40104-015-0013-4

**Published:** 2015-04-08

**Authors:** Qiufeng Zeng, Xueqin Huang, Yuheng Luo, Xuemei Ding, Shiping Bai, Jianping Wang, Yue Xuan, Zhuowei Su, Yonggang Liu, Keying Zhang

**Affiliations:** Institute of Animal Nutrition, Key Laboratory for Animal Disease-Resistance Nutrition of China Ministry of Education, Sichuan Agricultural University, Sichuan Ya’an, 625014 China; Adisseo Asia Pacific Pte Ltd, Singapore, 179360 Singapore

**Keywords:** Formulation specification, Growth, Phytase, Meat duck, Non-starch polysaccharides enzyme

## Abstract

**Background:**

Previous studies with broiler have shown dietary supplementation with multi-enzyme complex containing non-starch polysaccharides (NSP) degrading enzymes and phytase is efficient in releasing phosphorus (P), calcium (Ca), energy and amino acids from corn-soybean meal diets or corn-sorghum diets, hence compensating considerable levels of nutrients in formulation. Notwithstanding, such potentials have not been well defined in duck nutrition. Giving China being the largest duck producing country, we conducted this study to establish adequate specifications of major nutrients along with multi-enzyme complex to meat duck from day-old to slaughter, focusing on performance, utilization of nutrients and bone mineralization. Five dietary treatments were: Positive control (PC,T1 ): the nutrients concentration of diet for 1 to 14 d of age were apparent metabolizable energy(AME) 2,800 kcal/kg, crude protein (CP)19.39%, Ca 0.85%, available phosphorus (avP) 0.42%; for 15 to 35 d of age these parameters were AME 2,900 kcal/kg, CP 16.47%,Ca 0.76%,avP 0.38%; Negative control 1(NC1,T2), the AME and digestible amino acids (DAA) were reduced by 70 kcal/kg and 2.0%, avP and Ca by 1.0 g/kg from PC diet; Negative control 2( NC2,T4), the down-spec from PC diet was AME 100 kcal/kg, DAA 2.5%, avP 1.5 g/kg and Ca 1.2 g/kg; The enzyme complex was added at the same dosage (200 mL/ 1,000 kg) on NC1 (T3) and NC2 (T5) diets.

**Results:**

Comparing with the ducks fed on T1, T3 and T5 diets, the birds fed on NC2 diet showed the lowest (*P* < 0.05) body weight ( d 14 and 35), feed intake (d 35), tibia ash, Ca and P contents (d 14 and 35), and the utilization of nutrients (*P* < 0.05). The supplementation with the enzyme complex to the NC diets restored growth rate, utilization of nutrients and bone mineralization to the level of the PC diet, and increased AME by 60 kcal/kg and 117 kcal/kg, respectively for the NC1 and NC2 diets.

**Conclusion:**

These results suggest that down-spec AME by 100 kcal/kg, DAA by 2.5%, avP by 1.5 g/kg and Ca by 1.2 g/kg caused detrimental effects on duck performance compared with those fed on the PC diet, and these performance losses can be compensated by the addition of the multiple-enzyme complex.

## Background

Previous studies demonstrated single or multiple enzyme complex such as carbohydrases and phytase can improve utilization of dietary nutrients, thus decrease the cost of feeds in poultry production [1]. Carbohydrases can hydrolyze non-starch polysaccharides (NSP), and release the encapsulated nutrients from the cell walls, and reduce digesta viscosity [2,3]. Phytase is effective to release phosphorous through hydrolyzing phytate phosphorous compounds. The addition of 500, 1,000, and 1,500 units of phytase to basal diet for 7-d-old ducks released 0.453, 0.847, and 1.242 g inorganic P/kg of diet after 10 d, respectively[4]. Similarly, previous reports showed supplementation of phytase exerted positive effect on the utilization of energy, nitrogen (N) and amino acids (AA) in poultry diets [5-7]. A study showed that carbohydrases may facilitate phytase to access phytate [8]. Other studies also demonstrated that phytase and carbohydrases may exert synergistic effects in wheat-based diets [5,9] or an additive effect [10,11] in corn-based diets based on performance parameters. The response to a multi-enzyme complex seems to depend on several factors, including the type of diet, level of nutrients in the diet, dose of enzymes, age of birds, and even the genetic strain of the birds [12-14].

Francesch and Geraert [15] reported that the supplementation with a multi-enzyme complex containing NSP degrading enzymes and phytase is very efficient in compensating the down-spec from reduction of 2.0 g/kg available phosphorus (avP), 1.6 g/kg calcium (Ca), 85 kcal/kg apparent metabolizable energy (AME), and 3.0% digestible amino acids (DAA) of corn-soybean meal diets in broilers. However, there are studies showing significant differences between duck and broiler in terms of nutrients utilization [16]. By far, most enzyme efficiency studies were conducted with broilers, whereas information regarding ducks is scarce. China is the largest country for duck production, accounting for 3/4 of the global duck husbandry. How to improve feed efficiency of diets containing high levels of by-products remains on the top of priority in duck husbandry. The present study was designed to investigate a multi-enzyme complex containing NSP enzymes and phytase on the growth performance, nutrient utilization and bone mineralization of meat ducklings fed on diets consisting in corn, soybean meal and by-products.

## Methods

The experimental protocol used in this study was approved by the Institutional Animal Care and Use Committee of Sichuan Agricultural University.

### Experimental design, birds and diets

Exp. 1, the study was a 2 × 2 + 1 factorial design consisting in five treatments [2 levels of AME, DAA, Ca and avP reduction, with or without enzyme supplementation, plus a positive control]. The positive control (PC) diet was formulated to be adequate or to exceed requirements of NRC (1994) [17], NC1 (down-spec AME 70 kcal/kg, DAA 2.0%, avP 1.0 g/kg and Ca 1.0 g/kg) and NC2 (down-spec AME 100 kcal/kg, DAA 2.5%, avP 1.5 g/kg and Ca 1.2 g/kg). From days 1 to 14 and 15 to 35 of age, the PC diet provided avP 0.42% and 0.38%, NC1 provided 0.32% and 0.28%, and NC2 provided 0.27% and 0.23%, respectively, without or with the addition of an enzyme complex (Rovabio® Max LC, a concentrated liquid solution containing xylanase, β-glucanase and phytase as main activities, extracted from fermentation broths of *Penicillium funiculosum* and *Schizosaccharomyces pombe*, Adisseo France SAS). The liquid enzyme was applied after feed pelleting at a dose of 200 mL/1,000 kg feed to provide a minimum 1,100 visco-units of endo-β-1,4-xylanase, 100 units of endo-1,3(4)-β-glucanase, and 500 phytase units (FTU)/kg feeds. There were five dietary treatments replicated 7 times each of 16 ducks per replicate and allocated at random by blocks, according to the location in the experimental house. The feeding program consisted of 2 diets, starter diet supplied from d 1 to d 14, followed by a grower diet from d 15 to d 35 (Table 1). All diets were formulated following the amino acid profile established by our laboratory, with digestible lysine: digestible methionine : digestible threonine: digestible trytophan = 100:48:66:27 in starter diets; and 100:44:67:23; for grower diets [18]. Bentonite was used as diluent. Feeds were supplied in pelleted form with diameter 2 mm for the starter and 3 mm for the grower. The enzyme was sprayed in liquid form after pelleting on to cooled pellets and at a dilution rate of 1/20.

**Table 1:** **Diet composition of the positive and negative controls** (**as-fed basis)**

**Item**	**1 to 14 d**			**15 to 35 d**		
	**PC** ^**1**^	**NC1**	**NC2**	**PC**	**NC1**	**NC2**
Ingredients, %						
Corn	51.00	49.50	48.40	46.50	47.00	47.50
Rice bran	5.00	5.00	5.00	8.00	8.00	8.00
Wheat bran	8.00	9.00	10.00	15.00	15.00	15.00
Soybean oil	0.60	0.32	0.30	3.88	2.93	2.40
Soybean meal, CP 43%	20.00	19.00	18.50	8.00	7.60	7.60
Rapeseed meal, CP 36%	5.00	6.00	6.40	8.00	8.50	8.50
Cottonseed meal, CP 42%	5.00	5.00	5.00	6.00	6.00	6.00
L-Lysine HCl	0.240	0.233	0.235	0.270	0.255	0.250
DL-Methionine	0.211	0.203	0.202	0.126	0.118	0.116
Calcium carbonate	0.985	1.075	1.202	0.960	1.050	1.178
Dicalcium phosphate	1.490	0.885	0.580	1.175	0.580	0.280
L-Threonine	0.045	0.036	0.035	0.044	0.032	0.028
L-Tryptophan,10%	0.760	0.720	0.710	0.320	0.285	0.275
Bentonite	0.589	1.948	2.356	0.695	1.620	1.843
NaCl	0.40	0.40	0.40	0.40	0.40	0.40
Choline chloride	0.15	0.15	0.15	0.10	0.10	0.10
Vitamin premix^a^	0.03	0.03	0.03	0.03	0.03	0.03
Mineral premix^b^	0.50	0.50	0.50	0.50	0.50	0.50
Total	100.00	100.00	100.00	100.00	100.00	100.00
Calculated nutrients						
Metabolizable energy, kcal/kg	2,800	2,730	2,700	2,900	2,830	2,800
Crude protein, %	19.39	19.31	19.30	16.47	16.46	16.48
Calcium, %	0.85	0.75	0.73	0.76	0.66	0.64
Total phosphorous, %	0.77	0.68	0.64	0.79	0.69	0.64
Available phosphorous, %	0.42	0.32	0.27	0.38	0.28	0.23
Digestible Lysine, %	0.95	0.93	0.92	0.76	0.74	0.74
Digestible Methionine, %	0.45	0.45	0.44	0.33	0.33	0.32
Digestible Threonine, %	0.63	0.61	0.61	0.51	0.50	0.49
Digestible Tryptophan, %	0.26	0.25	0.25	0.17	0.17	0.17

^a,b^Vitamin and mineral content of the diets derived from the premixes were as follows (supplied per kg): vitamin A, 3,000 IU; vitamin D_3_, 1,500 IU; vitamin E, 7.5 IU; vitamin B_1,_ 0.6 mg; vitamin B_2,_ 4.8 mg; vitamin B_6_, 1.5 mg; vitamin B_12_, 0.009 mg; pantothenic acid, 7.5 mg; folic acid, 0.15 mg; niacin, 20 mg; Cu (CuSO_4_•5H_2_O), 8 mg; Fe (FeSO_4_•7H_2_O), 80 mg; Zn (ZnSO_4_•7H_2_O), 90 mg; Mn (MnSO_4_•H_2_O), 70 mg; Se (NaSeO_3_), 0.3 mg; I (KI), 0.4 mg.

^1^PC = positive control; NC1 = -70 kcal/kg, -2.0% digestible amino acids (DAA), -0.1 percentage point available P (avP), -0.1 percentage point Ca; NC2 = -100kcal/kg, -2.5% DAA, -0.15 percentage point avP, -0.12 percentage point Ca.

Exp. 2 was a subsequent metabolizable study of Exp. 1. At day 35, six ducklings were selected in line with the mean body weight of each treatment. A total of 30 ducklings (1 duckling per cage and 6 cages per treatment) were placed into individual metabolic cages (50 cm × 50 cm) in a room with temperature maintained at 22-25°C with continuous lighting. At day 39, each duckling was surgically fitted with a retainer ring [19] and fed on corresponding diets until restoration of feed intake and excretion, followed by feed withdrawal for 12 h. All ducklings were then provided with their respective corresponding diets and their faecal collection bags were installed. Excreta samples were collected for a period of 48 h. The excreta collected were immediately frozen at -20°C.

### Bird housing and management

Five hundred and sixty one-day-old male Cherry Valley ducks were used and distributed into 35 cages of 16 ducks per cage. All ducks were reared in cages (2.2  m × 1.2 m × 0.9 m) in a temperature and humidity controlled room with a 24 h constant light schedule and had free access to water and feed throughout the experimental period.

### Sampling and measurement

At the age of d 14 and d 35, birds and feed consumption were weighed by cage, body weight (BW) gain, feed intake (FI), feed to gain ratio (F/G) were then calculated. Seven ducklings per treatment (1 bird per pen) were randomly selected for blood sampling via the jugular vein. Serum was obtained by centrifuge at 3,000 rpm, 4°C for 10 min. All serum samples were stored -20°C. Contents of Ca, P and alkaline phosphatase (ALP) activity were measured by automated hematology analyzer (Yellow Springs Instrument Co., Inc., Yellow Springs, OH). Once blood was collected, birds were euthanized by cervical dislocation. Their left tibia was removed and stored at -20°C. After boiling/extraction, tibia ash, Ca and P contents were determined, according to the procedures of AOAC [20,21].

### Chemical analysis

Diet and dried excreta samples were ground to pass through 0.5 mm screen using a mill grinder. Duplicate proximate analyses were performed on diets and excreta samples. Dry matter(DM) analysis of samples was conducted by drying the samples in an oven at 105°C for 24 h. Energy content of the samples was determined by the adiabatic oxygen bomb calorimeter. Nitrogen, ester extract (EE), ash, Ca and total P contents of the samples were determined according to AOAC [21].

### Statistical analysis

To establish differences between PC and reduced nutrient diets, with and without enzyme supplementation, data were analyzed as a randomized complete block design with a one-way ANOVA using the GLM procedures of SAS [22]. To determine the main effects of level of nutrient reduction and enzyme supplementation response and their interaction, data without the PC were analyzed by a 2 × 2 factorial analysis of the variance. Statistical significance was set at *P* ≤ 0.05.

## Results and discussion

### Growth performance

The results of growth performance are shown in Table 2. Ducklings fed on NC2 diet had the lowest (*P* < 0.05) BW (d 14 and 35) and FI (d 35) compared to those fed on PC, NC1 with the enzyme and NC2 with the enzyme. The addition of the enzyme complex significantly increased (*P* < 0.05) BW and FI at days 14 and 35 to the level of PC diet, but no effect on F/G. Feed to gain ratio in NC1, NC1 with enzyme, NC2 and NC2 with enzyme diets was marked higher (*P* < 0.05) than that of the PC diet. Interaction between reduced dietary nutrient level and enzyme supplementation was detected (*P* <0.05) for BW at 14 d of age and FI during the whole experiment. These results suggest that NC1 nutrient levels (1 to 14 d, AME 2,730 kcal/kg, avP 0.32%, Ca 0.75%; 15 to 35 d, AME 2,830 kcal/kg, avP 0.27%, Ca 0.66%) were able to meet the growth requirement of the meat ducks; but NC2 nutrient level (1 to 14 d, ME 2,700 kcal/kg, avP 0.27%, Ca 0.73%; 15 to 35 d, ME 2,800 kcal/kg, avP 0.23%, Ca 0.64%) showed a negative effect on performance. Xie et al. [23] and Fan et al. [24] studied energy requirement of White Pekin ducklings from post-hatching to 21 d or 15 to 42 d by comparing 5 dietary energy levels (2,455, 2,603, 2,751, 2,902, 3,057 kcal/kg) or by 6 dietary energy levels (2,600, 2,700, 2,800, 2,900, 3,000, 3,100 kcal/kg), respectively, the results revealed that BW and FI seemed insensitive to dietary ME change within a range of 0 to 100 kcal/kg, whilst feed conversion did. We consider the poor performance of the ducks fed on NC2 diet in this study was resulted from avP deficiency as peviously reported by several authors [15,25,26].

**Table 2:** **Growth performance of ducks at days 14 and 35 of age** ^**1**^ **, Exp. 1**

**Item**	**Enzyme**	**14 d of age**			**35 d of age**		
		**BW, g**	**Feed intake, g**	**F/G, g/g**	**BW, g**	**Feed intake, g**	**F/G, g/g**
Treatment^2^							
T1:PC	-	695.1^a^	955.0^a^	1.48^a^	2395^a^	3950.1^a^	2.07
T2:NC1	-	676.8^ab^	970.1^b^	1.55^b^	2321^ab^	3908.7^a^	2.13
T3:NC1	+	690.7^a^	996.4^b^	1.56^b^	2371^ab^	3966.0^a^	2.12
T4:NC2	-	664.4^b^	956.2^a^	1.56^b^	2276^b^	3749.6^b^	2.09
T5:NC2	+	706.2^a^	1021.3^b^	1.56^b^	2391^a^	3978.1^a^	2.11
SEM		6.22	9.71	<0.01	25.37	43.22	0.02
* P*-Value		<0.01	<0.01	<0.01	<0.01	<0.01	0.239
*P*-Value of factorial analysis^3^							
NR^4^		0.803	0.589	0.615	0.625	0.160	0.355
Enzyme		<0.01	<0.01	0.626	<0.01	<0.01	0.782
NR × Enzyme		0.050	0.067	0.660	0.217	0.043	0.454

^a,b^Means in the same row, values with the same lowercase letter superscripts mean no significant difference (*P* > 0.05), different lowercase letter mean significant difference (*P* < 0.05).

^1^Data are means of 7 pens of 16 ducks.

^2^ PC = positive control; NC1 = − 70 kcal/kg, − 2.0% digestible amino acids (DAA), − 0.1 percentage point available P (avP), − 0.1 percentage point Ca; NC2 = − 100 kcal/kg, − 2.5% DAA, −0.15 percentage point avP, − 0.12 percentage point Ca.

^3^Using negative control treatments with or without enzyme supplementation.

^4^NR = Apparent metabolizable energy, digestible amino acid, available P and Ca reduction.

The supplementation with multiple-enzyme complex to the diets of chickens has been shown to liberate metabolizable energy from the otherwise unavailable sugars from NSP and P of the phytate molecule[2,15]. Meng and Slomiski [27] concluded that a combination of various carbohydrase enzyme preparations was more effective on cell wall degradation than were single enzymes, and that the extent of NSP degradation depended on the combination used. In the current study, the supplementation with both NSP-degrading enzymes and phytase to the reformulated diets (T3, AME 70 kcal/kg, avP 1.0 g/kg; T5, AME 100 kcal/kg and avP 1.5 g/kg) increased BW at 35 d of age by 1.4% and 5.7% (*P* < 0.05), respectively. This is consistent with the previous findings by Avila [28], who reported supplementing the same enzymes in the reformulated diets (AME 85 kcal/kg and 120 kcal/kg) increased BW gain by 1.93 and 2.01%, respectively. Similar results were also observed from broiler chicks with 14% increase in weight gain following the addition of a combination of xylanase, amylase, protease and phytase [10].

### Nutrient utilization

The effects of enzyme supplementation on nutrition utilization are summarized in Table 3. The down-spec of nutrients significantly decreased (*P* < 0.01) availability of CP and EE compared with the PC diet. Feeding NC2 diet reduced (*P* < 0.01) availability of DM, energy, CP, EE, ash, Ca and P compared to feeding PC and NC1 diet. The addition of the enzyme to the NC diets significantly improved (*P* < 0.05) energy, CP, EE, ash, Ca and P availability. Likewise, the enzyme addition to the NC2 diet significantly improved (*P* < 0.05) energy, DM, CP, EE, ash and P retention, but didn’t restore to the level of the PC (*P* < 0.05). Supplementing the enzyme complex to the NC1 diet increased AME by 60 kcal/kg, and in NC2 diet by 117 kcal/kg. Meng et al. [2] found improved digestibility of the NSP (from 6.3% without enzymes to 14.9% with enzymes) and AMEn (+2.3%) in a corn-soybean meal diet containing 9% total NSP. The function of phytase resulted in improvements in digestibility of dietary P and other minerals [5,26,29]. Carbohydrases can partially break-down cell wall matrix thus liberate more nutrients, and facilitate contact between endogenous digestive enzymes and their substrates, improving the overall utilization of nutrients in feedstuff [30]. This study showed that NSP-degrading enzymes and phytase were able to improve availability of energy, CP, EE, P, Ca and ash, thus boosting bird performance. This was well in line with Cowieson et al.[25,] and Cowieson and Adeola [10], who demonstrated that the performance of broiler chicks fed on low nutrient density corn-based diets returned to the level of birds fed on nutritionally adequate diet after supplementing with exogenous xylanase, amylase, protease and phytase by improving the digestibility of energy, DM, N, lipids, amino acids, Ca and P when broiler chicks fed on corn-soybean meal-based diets.

**Table 3:** **Apparent availability of nutrients and AME of test diets** ^**1**^ **, Exp. 2**

**Item**	**Enzyme**	**Energy %**	**DM** ^**2**^ **, %**	**CP, %**	**EE, %**	**Ash, %**	**Ca, %**	**P , %**	**AME, kcal/kg**
Treatment^3^									
T1:PC	-	84.7^a^	80.9^a^	81.0^a^	92.8^a^	40.4^a^	57.7^ab^	51.1^ab^	3254
T2:NC1	-	83.0^ab^	78.3^ab^	77.7^b^	87.8^b^	32.2^a^	54.6^b^	48.8^b^	3184
T3:NC1	+	84.9^a^	80.8^a^	77.9^b^	90.0^c^	42.7^a^	63.2^a^	56.4^a^	3244
T4:NC2	-	77.6^c^	71.9^c^	69.7^c^	85.4^d^	16.7^c^	43.9^c^	36.9^c^	2952
T5:NC2	+	80.8^b^	75.8^b^	76.6^b^	89.4^bc^	25.4^b^	64.6^a^	45.4^bd^	3069
SEM		1.00	0.65	0.91	0.49	1.73	2.63	1.40	20.1
*P*-Value		<0.01	<0.01	<0.01	<0.01	<0.01	<0.01	<0.01	<0.01
*P*-Value of factorial analysis^4^									
NR^5^		<0.01	<0.01	<0.01	0.014	<0.01	0.014	<0.01	<0.01
Enzyme		<0.01	<0.01	<0.01	<0.01	<0.01	<0.01	<0.01	<0.01
NR × Enzyme		0.261	0.341	<0.01	0.123	0.772	0.059	0.766	0.201

^a,b,c,d^Means in the same row, values with the same lowercase letter superscripts mean no significant difference (*P* > 0.05), different lowercase letter mean significant difference (*P* < 0.05).

^1^Data are means of 6 cages of 1 ducks.

^2^DM = dry matter; CP = crude protein; EE = ether extract; Ca = calcium; P = phosphorus; AME = apparent metabolizable energy.

^3^PC = positive control; NC1 = −70 kcal/kg, −2.0% digestible amino acids (DAA), −0.1 percentage point available P (avP), −0.1 percentage point Ca; NC2 = −100 kcal/kg, −2.5% DAA, −0.15 percentage point avP, −0.12 percentage point Ca.

^4^Using negative control treatments with or without enzyme supplementation.

^5^NR = Apparent metabolizable energy, digestible amino acid, available P and Ca reduction.

### Ca and P metabolism

At the age of 35 d, serum P content of the birds fed on NC2 diet decreased by 26.5% (*P* < 0.05) compared with PC diet (Table 4). The enzyme supplementation to NC1 increased the serum P by 35.1% (*P* < 0.05). The interaction of diets and enzyme supplementation was significant (*P* < 0.05) on serum P content. The effects of the enzyme supplementation on bone mineralization are summarized in Table 5. At 14 d, feeding NC1 diet significantly reduced the contents of tibia ash and Ca (*P* < 0.05), but not P. The enzyme supplementation to NC1 diet significantly improved tibia ash, P, Ca concentrations (*P* < 0.05) to the level of the PC group. At days 14 and 35, tibia ash, P, Ca concentrations of birds fed NC2 diet were lower (*P* < 0.05) compared with those on the PC diet. The enzyme addition to NC2 diet markedly improved these parameters (*P* < 0.05) to the level of the PC group, with an exception that tibia ash and P concentration at 14 d did not reach the level of the PC diet. Diet × enzyme interactions were detected for 35 d tibia ash and Ca concentration (*P* <0.05).

**Table 4:** **Serum calcium and phosphorus contents, and alkaline phosphatase activity of ducks at 14 d and 35 d of age** ^**1**^ **, Exp. 1**

**Item**	**Enzyme**	**14 d of age**			**35 d of age**		
		**Ca** ^**2**^ **, mmol/L**	**P, mmol/L**	**ALP, mmol/L**	**Ca, mmol/L**	**P, mmol/L**	**ALP, mmol/L**
Treatment^3^							
T1:PC	-	2.35	2.37	446.0	2.65	2.79^a^	474.2
T2:NC1	-	2.35	2.52	508.7	2.52	2.51^ab^	379.5
T3:NC1	+	2.22	2.6	474.6	2.52	2.29^ab^	383.7
T4:NC2	-	2.12	2.55	565.1	2.2	2.05^b^	382.1
T5:NC2	+	2.20	2.52	530.8	2.65	2.77^a^	420.5
SEM		0.17	0.16	29.07	0.16	0.17	56.45
*P*-Value		0.858	0.882	0.088	0.317	0.034	0.724
*P*-Value of factorial analysis^4^							
NR^5^		0.496	0.879	0.119	0.590	0.979	0.719
Enzyme		0.891	0.879	0.327	0.237	0.210	0.698
NR × Enzyme		0.585	0.718	0.997	0.237	0.027	0.755

^a,b^Means in the same row, values with the same lowercase letter superscripts mean no significant difference (*P* > 0.05), different lowercase letter mean significant difference (*P* < 0.05).

^1^Data are means of 7 ducks per treatment.

^2^Ca = Calcium, P = Phosphorus, ALP = Alkaline phosphatase.

^3^ PC = positive control; NC1 = −70 kcal/kg, −2.0% digestible amino acids (DAA), −0.1 percentage point available P (avP), −0.1 percentage point Ca; NC2 = −100 kcal/kg, −2.5% DAA, −0.15 percentage point avP, −0.12 percentage point Ca.

^4^Using negative control treatments with or without enzyme supplementation.

^5^NR = Apparent metabolizable energy, digestible amino acid, available P and Ca reduction.

**Table 5:** **Tibia ash, calcium and phosphorus contents of ducks at 14 d and 35 d of age** ^**1**^ **, Exp. 1**

**Item**	**Enzyme**	**14 d of age**			**35 d of age**		
		**Ash,%**	**Ca** ^**2**^ **, %**	**P,%**	**Ash,%**	**Ca, %**	**P,%**
Treatment^3^							
T1:PC	-	44.22^a^	16.13^a^	10.19^ab^	53.77^a^	20.51^a^	7.84^a^
T2:NC1	-	41.51^b^	14.75^b^	10.07^ab^	53.86^a^	20.67^a^	7.60^a^
T3:NC1	+	43.31^ab^	15.97^a^	10.31^a^	53.84^a^	20.69^a^	8.04^b^
T4:NC2	-	37.40^c^	13.55^c^	9.44^c^	50.10^b^	18.96^b^	6.98^c^
T5:NC2	+	40.11^b^	14.47^b^	9.93^b^	52.66^a^	20.39^a^	7.62^a^
SEM		0.73	0.28	0.10	0.55	0.18	0.14
*P*-Value		<0.01	<0.01	<0.01	<0.01	<0.01	<0.01
*P*-Value of factorial analysis^4^							
NR^5^		<0.01	<0.01	<0.01	<0.01	<0.01	<0.01
Enzyme		<0.01	<0.01	<0.01	0.032	<0.01	<0.01
NR × Enzyme		0.611	0.568	0.545	0.029	<0.01	0.277

^a,b ,c^Means in the same row, values with the same lowercase letter superscripts mean no significant difference (*P* > 0.05), different lowercase letter mean significant difference (*P* < 0.05).

^1^Data are means of 7 ducks per treatment.

^2^Ca = Calcium, P = Phosphorus.

^3^ PC = positive control; NC1 = −70 kcal/kg, −2.0% digestible amino acids (DAA), −0.1 percentage point available P (avP), −0.1 percentage point Ca; NC2 = −100 kcal/kg, −2.5% DAA, −0.15 percentage point avP, −0.12 percentage point Ca.

^4^Using negative control treatments with or without enzyme supplementation.

^5^NR = Apparent metabolizable energy, digestible amino acid, available P and Ca reduction.

The dietary nutrient reduction resulted in decrease in bone mineralization, in terms of serum P and Ca content and ALP activity, and tibia ash, P, and Ca contents, compared with the birds fed on adequate level of nutrients. Serum mineral concentration is often used as an important indicator of mineral status of birds. In this study, serum P concentration at 35 d of age decreased by 26.5% when the duckings fed on NC2 diet (avP: 0.27% for d 1 to 14; 0.23% for d 15 to 35). Viveros et al. [26] reported that decreasing avP levels in broiler diet could decrease plasma P level by 27.5% in 6 weeks. The enzyme supplementation to NC2 diet increased serum P concentration to the level of the PC diet. These results were well in line with previous studies [31,32].

Bone mineral concentration may be more sensitive than performance in estimating mineral deficiency. The requirement of avP and Ca for maximum P, Ca and ash deposition in tibia was greater than for adequate growth performance [15,26]. Similar results were observed in the current study, in which the enzyme supplementation increased bone mineralization of ducklings fed on both NC1 and NC2 diets, with a greater response observed from the birds fed on the most avP and Ca inadequate diets. With reduction on avP and Ca contents (avP 1.5 g/kg and Ca 1.2 g/kg), supplemental enzymes restored performance in full, but insufficient to restore bone mineralization to that of the PC. Francesch et al. [15], who investigated the same multiple-enzyme complex in corn-soybean meal diets for broilers, found significantly differences in bone ash mineralization at 43 d between the NC and PC groups, as well as with the NC diet with the multiple-enzyme complex. Dilger et al. [29] found that overall tibia ash improved with phytase addition (P < 0.05) during a 42-d period in which broiler chickens were fed on an NC diet (with the starter and grower diets containing 2.4 and 1.8 g/kg non-phytate P, respectively).

In conclusion, results of the present experiment indicate that the lower nutrient density diets resulted in a reduction in growth performance, nutrient utilization and bone mineralization of meat ducklings which can be restored by supplementation of exogenous enzyme complex, and that the multi-enzyme complex has the potential to compensate AME 100 kcal/kg, DAA 2.5%, av P 1.5 g/kg and Ca 1.2 g/kg in basal diet consisting in corn, soybean meal and by-products.

## Abbreviations

ALP: Alkaline phosphatase
AME: Apparent metabolizable energy
avP: Available phosphorus
BW: Body weight
Ca: Calcium
DAA: Ddigestible amino acid
DM: Dry matter
EE: Ester extract
FI: Feed intake
F/G: Feed to gain ratio
NC: Negative control
NSP: Non-starch polysaccharides
PC: Positive control
N: Nitrogen
